# Increased risk of major depression subsequent to a first-attack and non-infection caused urticaria in adolescence: a nationwide population-based study

**DOI:** 10.1186/1471-2431-14-181

**Published:** 2014-07-11

**Authors:** Chia-Lun Kuo, Chi-Yen Chen, Hui-Ling Huang, Wen-Liang Chen, Hua-Chin Lee, Chih-Yu Chang, Chu-Chung Chou, Shinn-Ying Ho, Han-Ping Wu, Yan-Ren Lin

**Affiliations:** 1 Tsao-Tun Psychiatric Center, Nan-Tou, Taiwan; 2 Department of Biological Science and Technology, National Chiao Tung University, Hsinchu, Taiwan; 3 Institute of Bioinformatics and Systems Biology, National Chiao Tung University, Hsinchu, Taiwan; 4 Department of Emergency Medicine, Changhua Christian Hospital, Changhua, Taiwan; 5 Department of Pediatrics, Buddhist Tzu Chi General Hospital, Taichung Branch, 66 Section 1, Fongsing Road, Taichung 42743, Taiwan; 6 School of Medicine, Chung Shan Medical University, Taichung, Taiwan

**Keywords:** Non-infection caused urticaria, Major depression, Adolescent, Pediatric, Hazard ratio

## Abstract

**Background:**

Non-infection caused urticaria is a common ailment in adolescents. Its symptoms (e.g., unusual rash appearance, limitation of daily activities, and recurrent itching) may contribute to the development of depressive stress in adolescents; the potential link has not been well studied. This study aimed to investigate the risk of major depression after a first-attack and non-infection caused urticaria.

**Methods:**

This study used the Taiwan Longitudinal Health Insurance Database. A total of 5,755 adolescents hospitalized for a first-attack and non-infection caused urticaria from 2005 to 2009 were recruited as the study group, together with 17,265 matched non-urticarial enrollees who comprised the control group. Patients who had any history of urticaria or depression prior to the evaluation period were excluded. Each patient was followed for one year to identify the occurrence of depression. Cox proportional hazards models were generated to compute the risk of major depression, adjusting for the subjects’ sociodemographic characteristics. Depression-free survival curves were also analyzed.

**Results:**

Thirty-four (0.6%) adolescents with non-infection caused urticaria and 59 (0.3%) non-urticarial control subjects suffered a new-onset episode of major depression during the study period. The stratified Cox proportional analysis showed that the crude hazard ratio (HR) of depression among adolescents with urticaria was 1.73 times (95% CI, 1.13-2.64) than that of the control subjects without urticaria. Moreover, the HR were higher in physical (HR: 3.39, 95% CI 2.77-11.52) and allergy chronic urticaria (HR: 2.43, 95% CI 3.18-9.78).

**Conclusion:**

Individuals who have a non-infection caused urticaria during adolescence are at a higher risk of developing major depression.

## Background

Urticaria is a common disease in children and is estimated to affect 15-25% of people at some point in their lives [1-3]. Symptoms of urticaria (e.g., recurrent itching, generalized wheals and sleep disturbances) can persist for several days to months and are a significant source of patient stress [1-8]. There are many etiologies of urticaria in children, including foods, infections, physical contact, temperature changes, and idiopathic causes [4,9-13]. Moreover, fruits and insect venom have also been reported to induce allergic reactions or urticaria in childhood [14,15]. Among children with urticaria, simple infections have been associated with the majority of acute episodes [2,9]. However, the stress and urticarial symptoms caused by simple infections are usually transient, particularly when patients are protected from the source of infection.

Non-infection caused urticaria may result in prolonged or recurrent episodes of urticaria. A first-attack episode of non-infection caused urticaria can impose limitations on the lifestyles of patients and their families. For example, patients who have suffered from food-induced urticaria in the past due to peanut allergies would subsequently need to eliminate their exposure to peanut-based products. Thus, an allergy-related event of this type is stressful to the patient and is also likely to have an impact on the entire family’s dietary choices. In addition to the unusual-looking rash, the adolescent’s interpersonal relationships with peers might result in limitations of daily activities as the severity of physical urticaria can be increased by exercise, skin contact and even sunlight [5,10,16]. One previous study reported that 43% of adult patients with urticarial dermographism experienced an impact on their quality of life and psychosocial stress [7]. Other specific dermatologic disorders have also been reported to be risk factors for the development of psychiatric problems in adulthood [6-8,17]. Psoriasis and atopic dermatitis can result in personality changes or depressive symptoms because of sleep disturbances or health-related impairment to quality of life [6,17]. Similarly, urticaria in adults has been reported to increase the likelihood of anxiety and depression [8] However, the relationship between psychiatric problems and pediatric non-infection caused urticaria is unclear. To our knowledge, urticaria-related depression in adolescents has never been studied. It is well known that adolescence is a unique developmental period marked by processes such as increased cognitive abilities and physical changes. During this period, adolescents may be vulnerable to the development of various mental and physical conditions [18]. Therefore, we suspect that a first-attack episode of non-infection caused urticaria might increase the likelihood of suffering a subsequent episode of new-onset major depression. In this study, we aimed to provide insights into urticaria-related major depression in adolescents.

## Methods

### Database

We used the Longitudinal Health Insurance Database (LHID) as the data source for this study. The LHID is derived from medical claims data available to the Bureau of National Health Insurance and provided to scientists in Taiwan for research purposes. The government of Taiwan launched its National Health Insurance (NHI) program in 1995 to provide affordable health care for all residents of Taiwan. As of 2007, over 98% of Taiwan's population was enrolled in this program. The LHID includes original data from one million people. The data in this study were randomly sampled from the period between 2005 and 2009. There were no significant differences in the gender or age distributions or the average payroll-related insurance premium rate between the people in the LHID and all NHI enrollees. The LHID also provides a valuable opportunity for researchers to evaluate medical service use since 1995. The details regarding how the database was generated are published online by the Taiwan National Health Research Institutes.

### Ethics statement

This study was exempt from a full review by the Institutional Review Board of Changhua Christian Hospital (permission code: 121007) because the data set consisted of de-identified secondary data that were released for research purposes without restrictions. In addition, this manuscript has adhered to the strengthening the reporting of observational studies in epidemiology (STROBE) guidelines.

### Study setting and population

This is a retrospective cohort study. During the period from January 1, 2005, to December 31, 2009, data were collected from the LHID for two patient groups, the study group and the control group. The study group was defined as adolescents who suffered non-infection caused urticaria. The control group was defined as adolescents who did not suffer any urticaria. We designated the first hospitalization for urticaria treatment during this period as the index hospitalization. In this study, the study group (with non-infection caused urticaria) and the control group (without any urticaria) were both followed for one year. The likelihood of suffering a new-onset episode of major depression during the one-year follow-up period was analyzed for the two groups.

### Inclusion criteria

#### Definition of patients with non-infection caused urticaria

Patients who were diagnosed with a principal diagnosis of urticaria using the International Classification of Diseases, 9th Revision, Clinical Modification codes (ICD-9-CM; code 708.0 to 708.9) were included in the study provided they did not have a co-diagnosis of infection (using ICD-9 codes) [19,20] and did not receive any antibiotic agents (including oral, injectable and ear-drop forms) at the time of their urticarial attack, or for 7 days before or after their attack. For those non-infection caused urticaria patients whom continuous treatment records were available, chronic urticaria was defined as urticarial symptoms that lasted for more than 6 weeks [1]. The possible etiologies of chronic urticaria were mainly classified as physical or allergic in nature. In this study, the chronic urticaria patients were included in the main study group.

#### Definition of patients with major depression

Patients were included in the study if they were diagnosed by a psychiatrist with major depression as the principal diagnosis using the ICD-9-CM codes 296.2 and 296.3. The diagnosis of major depression adhered to the definitions and criteria in the Diagnostic and Statistical Manual of Mental Disorders (DSM) - IV published by the American Psychiatric Association [21]. Bipolar depression, affective disorder substance-related depression, postpartum depression and depressive disorder were also not included as our primary outcomes because the principles of their diagnoses and their clinical presentations are different compared with major depression. Affective disorder may include manic attacks, and depressive disorder has only some of the same symptoms as major depression.

### Exclusion criteria

Patients who had been diagnosed with any form of urticaria or depression prior to their index hospitalization (first-attack of non-infection caused urticaria) were not included.

### Quality control for potential icd-9 over coding and treatments

To ensure that the medical resources provided by the government-supported NHI program were not over-used by the treating hospitals or patients, the diagnosis, treatments, and medications for each patient were randomly and routinely inspected by specialists. Over-treatment or over-coding in ICD-9 was not permitted and can result in fines.

### Study protocol

Our study group included 5,755 non-infection caused urticaria patients (adolescents 13 to 18 years of age). The control group was selected from the remaining NHI beneficiaries registered in the LHID. We then randomly selected 17,265 control patients (three control patients for each urticaria patient) who were matched to the study group by gender, age and years of index healthcare use for further analysis. A total of 23,020 adolescents were included in this study.

### Data analysis

The SAS 9.2 statistical package was used to perform the study analyses (SAS Institute Inc., Cary, NC, USA). We used the SAS program to select the study and control groups. Each patient (n = 23,020) was tracked for one year after his/her index hospitalization to identify subjects who developed new-onset major depression. The results of the descriptive analyses of the independent variables including patient characteristics, demographics, personal allergy histories, family history (parents/brothers/sisters) of affective disorders are reported as percentages or as the mean ± standard deviation (SD). The *X*^*2*^ test was used to compare the differences between the study and control groups with regard to demographics, including socioeconomic level (i.e., monthly income of the patient and guardian > $1000 USD, $601-1000 USD or < $600 USD), the degree of urbanization in their cities of residence (levels 1 to 4), the geographical location of the patient’s residence (northern, central, southern, and eastern Taiwan), and the personal history of allergic diseases (allergic rhinitis, asthma, and atopic dermatitis). The degree of urbanization was defined by population and certain development-related conditions. Level 1 urbanization was defined as a population over 1,250,000 people with specific political, economic, cultural, and metropolitan development. Level 2 urbanization was defined as a population between 500,000 and 1,250,000 with political, economic and cultural development serving an important role. Levels 3 and 4 were defined as a population between 150,000 and 500,000 and less than 150,000 people, respectively.

Furthermore, the crude hazard ratio (HR) was calculated by creating stratified Cox's proportional hazards models (stratified by age), which were implemented in the study and control groups to analyze the risk of experiencing a new-onset of depression. In addition, the variables that were related and unrelated to the occurrence of depression among the urticarial patients were further analyzed using the *X*^*2*^ test. These variables included gender, age, socioeconomic level, the urbanization level in the city of residence, geographic regions, history of allergic diseases, family history of affective disorders, urticaria treatment with corticosteroids (oral and injection forms) and the mean number of hospital visits (for urticaria treatment). Age groups and the causes of chronic urticaria were also analyzed to identify interactions between these parameters (case/control groups, demographics and allergy histories) with a Cox proportional hazards model. The adjusted HR was analyzed after adjusting for allergic rhinitis, asthma, atopic dermatitis, family history of affective disorders, urticaria treatment with corticosteroids (oral and injection forms), geographic regions, socioeconomic level, and the urbanization level of their cities of residence. We used the Kaplan-Meier method and the log-rank test to estimate survival curves and to compare the one-year depression-free survival rate among urticaria patients versus patients in the control group. Finally, in the control group, chronic conditions (e.g., allergic rhinitis, asthma, attention deficit disorder, atopic dermatitis, hypertension, epilepsy, diabetes, congenital heart diseases, cerebral palsy and cancer) were identified that might increase the risk for affective or anxious disorders [22-31].

## Results

### Demographics of patients with non-infection caused urticaria

The characteristics and personal histories of allergic diseases of the study patients (with non-infection caused urticaria, n = 5,755) and controls (without urticaria; n = 17,265) are presented in Table 1. Among the urticarial patients, the 16- to 18-year-old age group was the most represented (54%). Compared with the control patients, more urticarial patients lived in southern Taiwan. The urticarial patients also had a significantly higher prevalence of allergic diseases than the control group (asthma, atopic dermatitis and allergic rhinitis, all *p* < 0.05).

**Table 1:** Characteristics and personal histories of adolescents with non-infection caused urticaria and control patients

	**Adolescents with non-infection caused urticaria (n = 5,755)**		**Control patients (n = 17,265)**		
	**No.**	**%**	**No.**	**%**	***p*-value**
Gender					1.000
Male	2,657	46.2	7,971	46.2	
Female	3,098	53.8	9,294	53.8	
Mean age (y/o) (Mean ± SD)	16.2 ± 1.7		16.2 ± 1.7		1.000
Age group (y/o)					1.000
13-15	2,645	46	7,935	46	
16-18	3,110	54	9,330	54	
Economic level of family (monthly income) (USD$)					0.246
<600	1,622	28.2	4,737	27.4	
601 ~ 1,000	3,125	54.3	9,349	54.2	
>1,000	1,008	17.5	3,179	18.4	
Urbanization					0.622
1 (most)	1,313	22.8	4,068	23.6	
2	703	12.2	2,102	12.2	
3	1,783	31	5,225	30.3	
4	1,956	34	5,870	34	
Geographic regions of Taiwan*					0.036
Northern	2,659	46.2	8,105	46.9	
Central	1,371	23.8	4,306	24.9	
Southern	1,553	27	4,401	25.5	
Eastern	172	3	453	2.6	
Asthma history*					0.004
Yes	2,144	37.3	6,093	35.3	
No	3,611	62.7	11,172	64.7	
Atopic dermatitis history*					<0.001
Yes	394	6.8	771	4.5	
No	5,361	93.2	16,494	95.5	
Allergic rhinitis*					<0.001
Yes	2,886	50.1	8,068	46.7	
No	2,869	49.9	9,197	53.3	
Family history of affective disorders	339	5.9	932	5.4	0.151

*Significant differences.

### Depression likelihood based on the crude HR

During the one-year follow-up period, the incidence of major depression was significantly higher among the urticaria patients than among the control patients. In this study, 0.6% (n = 34) of patients suffered a new onset of major depression after an episode of urticaria, whereas the corresponding percentage was only 0.3% (n = 59) in the control group. The population attributable risk for major depression between non-infection caused urticaria exposed and non-exposed was 0.3% (0.6% - 0.3% =0.3%). The stratified Cox proportional hazard analysis showed that the study group had a crude HR that was 1.73-times greater than that of the control group (95% CI, 1.13-2.64, *p* < 0.05). The chronic conditions that might the increase risk of affective or anxious disorders in the control group included allergic rhinitis (n = 8,068, 46.7%), asthma (n = 6,093, 35.3%), attention deficit (n = 862, 5.0%), atopic dermatitis (n = 771, 4.5%), hypertension (n = 401, 2.3%), epilepsy (n = 345, 2.0%), diabetes (n = 271, 1.6%), congenital heart diseases (n = 92, 0.4%), cerebral palsy (n = 32, 0.2%) and cancer (n = 16, 0.1%).

### Pharmacological treatment (corticosteroids)

There were 2,473 patients who had ever received corticosteroids for their urticaria. The prevalence of major depression was higher in the group treated with corticosteroids (n = 31, 1.3%) compared with the group without corticosteroids (n = 3, 0.1%) (*p* < 0.05). Moreover, in the adjusted model, the urticarial patients who had ever received corticosteroids were more likely to experience new-onset major depression compared with the control group patients (HR = 1.89; 95% CI = 1.23-2.87; *p* < 0.05) (Table 2).

**Table 2:** The adjusted-effect estimates for urticaria

**Variables**	**Occurrence of new-onset major depression**	
	**HR**	**95% CI**
Adolescents with non-infection caused urticaria	1.73	1.13-2.64
Control*	1.00	1.00
Mode 1		
Adolescents with non-infection caused urticaria	1.72	1.13-2.63
Control*	1.00	1.00
Mode 2		
Adolescents with non-infection caused urticaria	1.71	1.12-2.61
Control*	1.00	1.00
Mode 3		
Adolescents with non-infection caused urticaria	1.85	1.17-2.93
Control*	1.00	1.00
Mode 4		
Adolescents with non-infection caused urticaria	1.89	1.23-2.87
Control*	1.00	1.00

*Reference group.

Mode 1: Adjusted by demographics (i.e., economic level of family, degree of urbanization and geographical location).

Mode 2: Adjusted by personal allergy histories (i.e., allergic rhinitis, asthma and atopic dermatitis).

Mode 3: Adjusted by family history of affective disorders.

Mode 4: Adjusted by treatment (i.e., corticosteroids).

### Characteristics that are associated with the occurrence of new-onset major depression in patients with non-infection caused urticaria (n = 5,755)

For the urticarial patients, we found that major depression was more predominant in the 16- to 18-year-old age group and history of asthma (both *p* < 0.05) (Table 3). Gender and the mean number of hospital visits were not significantly associated with the onset of depression. After adjusting for the patients’ demographics and histories of allergic diseases, the urticarial patients were still more likely to experience new-onset major depression than the control group patients (Table 2). Of all, there were 217 chronic urticaria patients (physical cause, n = 82 and allergy cause, n = 135). Three of 82 (3.7%) physical caused urticaria patients and 2 of 135 (1.5%) allergy caused urticaria patients suffered major depression. The crude HR of depression of physical causes was 3.39 times (95% CI 2.77-11.52) and of allergy causes was 2.43 (95% CI 3.18-9.78) times greater than that of the control subjects without urticaria (both p < 0.05). Urticaria-related depression was significantly more severe than the depression observed in the control group. Finally, the age groups and the causes of chronic urticaria did not exhibit significant interactions with the case/control groups, demographics or allergy histories.

**Table 3:** Characteristics associated with the occurrence of new-onset major depression in adolescents with non-infection caused urticaria

	**Adolescents with non-infection caused urticaria (n = 5,755)**		**Control patients (n = 17,265)**	
	**New-onset major depression occurrence (n = 34) No. (%)**	**p –value**	**New-onset major depression occurrence (n = 59) No. (%)**	**p –value**
Gender				
Male	15 (44.1)	0.475	22 (37.3)	0.192
Female	19 (55.9)		37 (62.7)	
Age group (y/o)*				
13-15	8 (23.5)	0.006	19 (32.2)	0.037
16 ~ 18	26 (76.5)		40 (67.8)	
Economic level of family (monthly income) (USD$)				
<600	15 (44.1)	0.095	21 (35.6)	0.264
601 ~ 1,000	13 (38.2)		26 (44.1)	
>1,000	6 (17.6)		12 (20.3)	
Urbanization				
1 (most)	5 (14.7)	0.251	14 (23.7)	0.123
2	7 (20.6)		3 (5.1)	
3	13 (38.2)		25 (42.4)	
4	9 (26.5)		17 (28.8)	
Geographic regions of Taiwan				
Northern	15 (44.1)	0.660	27 (45.8)	0.543
Central	11 (32.4)		12 (20.3)	
Southern	7 (20.6)		17 (28.3)	
Eastern	1 (2.9)		3 (5.1)	
Asthma history*	19 (55.9)	0.020	23 (39.0)	0.589
Atopic dermatitis history	3 (8.8)	0.415	4 (6.8)	0.337
Allergic rhinitis	16 (47.1)	0.425	28 (47.5)	1.000
Family history of affective disorders*	27 (79.4)	0.002	43 (72.9)	0.003
Urticaria treatment with corticosteroids*	31 (91.1)	<0.001	-	
Mean number of hospital visits (to treat urticaria)	1.6 ± 1.2	0.578	-	

*Characteristics associated with depression in study group.

### Depression-free survival curves for patients

**Figure 1:**
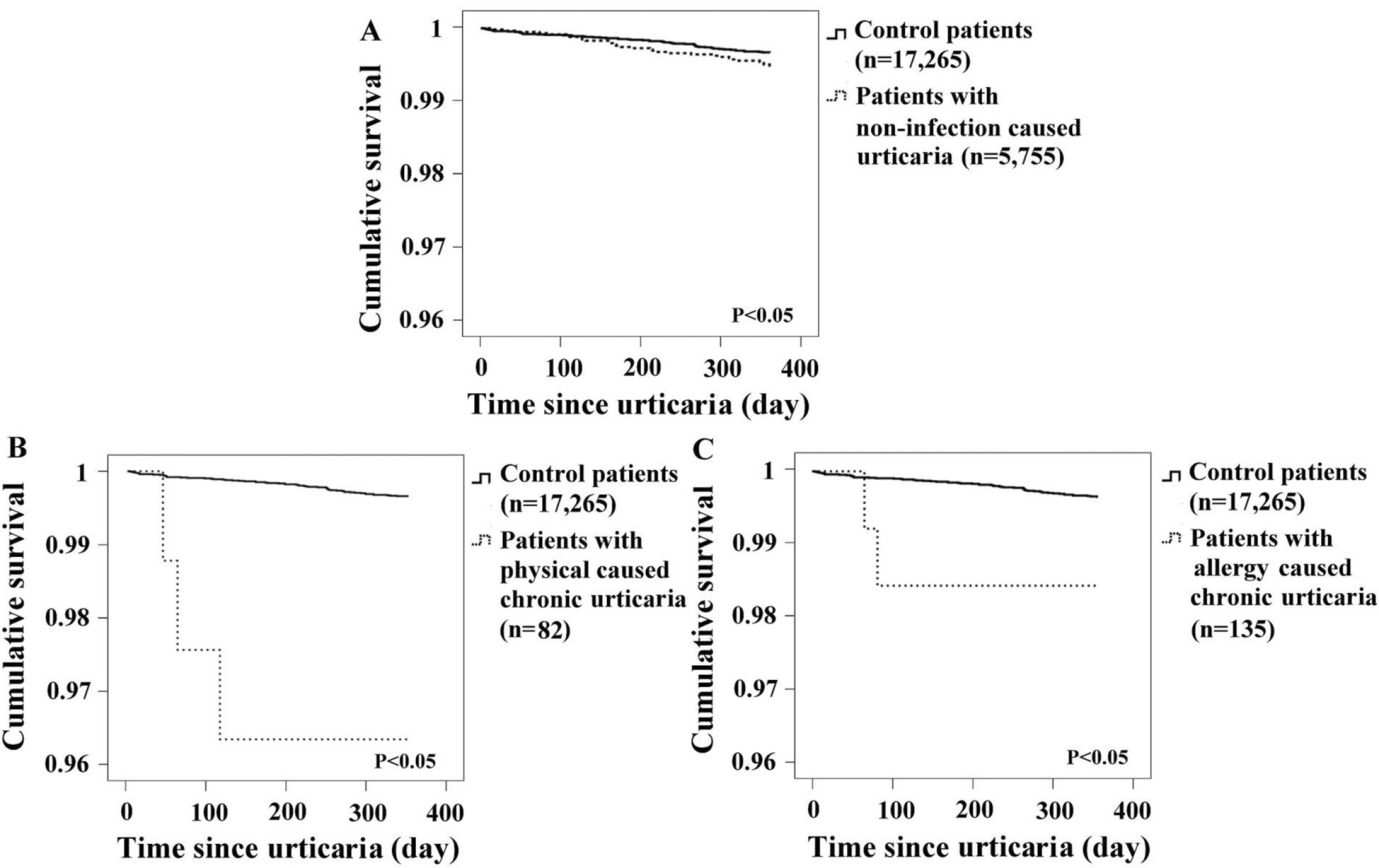
**Depression-free survival curves.** Depression-free survival curves for the **(A)** non-infection caused urticaria, **(B)** physically induced chronic urticaria and **(C)** allergy-induced chronic urticaria patients during the 1-year follow-up period (all p < 0.05). The curves for all patients with non-infection caused urticaria were similar to those in the small cohorts of patients with chronic allergic (n = 135) or physical caused urticaria (n = 82).

The depression-free survival curves for the urticaria and control patients during the study period are shown in Figure 1. We noted that the urticaria patients had significantly shorter one-year depression-free survival times than the control patients (all *p* <0.05).

## Discussion

In this one-year follow-up study, we noted that non-infection caused urticaria significantly caused the subsequent major depression. The stratified Cox proportional analysis showed that the crude hazard ratio of depression among adolescents with urticaria was 1.73 times (95% CI, 1.13-2.64) than that of the control subjects without urticaria (p < 0.05). Although the sample size of chronic urticaria is small, we still noted that physical (crude HR: 3.39 95%, CI 2.77-11.52) or allergy caused (crude HR: 2.43, 95% CI 3.18-9.78) chronic urticaria have a higher risk of suffering depression than control group. Some factors that might potentially influence the occurrence of depression, including baseline personal allergic histories, economic conditions of the family, and geographic regions were adjusted. Non-infection caused urticaria still significantly increased the hazard ratio of major depression. Finally, the age groups and the causes of chronic urticaria did not exhibit significant interactions with the case/control groups, demographics or allergy histories.

The incidence of major depression occurrence of adolescents with non-infection caused urticaria was low (0.6%); however, it was still significantly higher than adolescents without any urticaria (0.3%). Among these adolescents with non-infection caused urticaria, two patient characteristics that were most likely to associate a subsequent episode of depression. First, the older adolescent group (aged 16 to 18 years) was more predominant than the younger adolescents (aged 13 to 15 years). Second, a history of asthma was the other factor that associates with a subsequent episode of depression. Data of the Table 3 showed that asthma history only significantly associated with depression occurrence in study group (but not in control group). Because some children with a history of asthma have already experienced some limitations in their daily activities (e.g., avoiding microorganisms from pets, abstaining from vigorous exercise and avoiding cold environments and allergenic foods) [32-37], the experience of new-onset urticaria could further extend these limitations and increase life stresses. In addition to only treat the urticarial symptoms, we suspect that the altered physical appearance (caused by recurrent rashes) and limited social activities (avoiding exercise and skin contact that might increase pruritus) might further contribute to the depressive mood of adolescents. Life stresses, including poor quality of life, social phobia, severe itching, and sleep disturbances, are usually present in most adult patients who have suffered from certain prolonged dermatologic diseases [7,8,17]. However, the association between dermatologic diseases and psychologic problems in adolescents has not been thoroughly addressed. It is well known that adolescent development represents a time of increased susceptibility to stress that is marked by increased vulnerability [38]. During adolescence, the brain demonstrates a high level of plasticity and can be positively or negatively affected by the environment [18,39]. Because elevated life or social stresses have been demonstrated to cause depressive episodes in adolescence [38,40,41]. Therefore, early psychiatric care to prevent depression in adolescents with urticaria may be important.

In addition, affective disorders have been demonstrated to be increased by chronic autoimmune/allergy conditions that increase life stress, social phobia or even chronic central nerve inflammatory reactions [40-42]. For example, autoimmune diseases (including atopic dermatitis and systemic lupus erythematosus) and allergic diseases (including allergic rhinitis and asthma) could increase the risk of depression or bipolar disorders [28,30,31,43]. Because these autoimmune/allergy diseases are strongly genetic in origin and have been demonstrated to potentially develop as heritable diseases [44], identifying the family history is important. Identification of autoimmune/allergy diseases in parents might help family physicians in the early detection of children with silent symptoms. The risks of suffering from affective disorders might be decreased by the early control of their chronic autoimmune/allergy conditions.

### Limitations

Potential ICD-9 over- or miscoding was an inherent limitation of this database study. The codes sent to the National Health Database were only made by attending physicians in outpatient/emergency departments. According to the Taiwan’s law, all codes must be made and confirmed by the treating physicians. Because major depression requires more clearly defined diagnostic criteria, which we discussed in the methods section, all cases of major depression were only diagnosed by psychiatrists. Urticaria is a common disease in dermatology, pediatric and even rheumatology outpatient departments; therefore, we did not limit the urticaria diagnosis to diagnoses made by dermatologists. Our procedure for separating the different divisions of treating physicians was to trace who required treatment payments from the government. Excluding patients who had a co-diagnosis of infection 7 days before or after their urticaria attack might represent an over exclusion. However, because upper airway infection and acute gastroenteritis are the most commonly reported etiologies of infection caused urticaria [9], we established this exclusion criterion to account for possible incubation periods. Finally, because corticosteroids are typically recommended to treat patients with more itching or recurrent urticaria [45], the results could have potentially been influenced by the condition of urticaria.

## Conclusion

Individuals who have a non-infection caused urticaria during adolescence are at a higher risk of developing major depression than those without urticaria.

## Abbreviations

HR: Hazard ratio; LHID: Longitudinal Health Insurance Database; NHI: National Health Insurance; STROBE: Strengthening the reporting of observational studies in epidemiology ICD-9-CM, International Classification of Diseases, 9th Revision, Clinical Modification codes; DSM: Diagnostic and Statistical Manual of Mental Disorders; SD: Standard deviation; CI: Confidence interval.

## Competing interests

There are no conflicts of interest related to this study.

## Authors’ contributions

C-LK, C-YC, H-PW and Y-RL conceived the study. Y-RL, W-LC, H-CL, C-YC and C-CC managed the data, including quality control. S-YH, Y-RL, W-LC and H-LH provided statistical advice on study design and analyzed the data. H-PW, Y-RL and S-YH chaired the data oversight committee. C-LK and C-YC drafted the manuscript, and all authors contributed substantially to its revision. H-PW, S-YH and Y-RL take responsibility for the paper as a whole. All authors read and approved the final manuscript.

## Pre-publication history

The pre-publication history for this paper can be accessed here:

http://www.biomedcentral.com/1471-2431/14/181/prepub
